# CHARACTERIZING THE CIRCUMSTANCES OF FALLS AMONG COMMUITY-DWELLING OLDER ADULTS

**DOI:** 10.1093/geroni/igae098.3362

**Published:** 2024-12-31

**Authors:** Abigail Kehrer-Dunlap, Rebecca Bollinger, Audrey Keleman, Beau Ances, Susan Stark

**Affiliations:** Washington University in St. Louis, St. Louis, Missouri, United States; Washington University in St. Louis, St. Louis, Missouri, United States; Department of Veterans Affairs, St. Missouri, United States; Washington University in St. Louis, St. Louis, Missouri, United States; Washington University in St. Louis, St. Louis, Missouri, United States

## Abstract

Falls are the leading cause of injury, institutionalization, and injury-related mortality among older adults. As the older adult population is expected to grow exponentially in the next decade, the cost and consequences of falls will continue to rise. While promising fall prevention interventions exist, the death rate due to falls continues to climb. Limited population health effectiveness of these interventions may be attributable to a poor understanding of contributing factors to falls, such as the environmental and contextual circumstances in which falls occur. The objective of this study was to characterize the circumstances of falls among community-dwelling older adults. We completed monthly, prospective fall monitoring with 350 cognitively normal (Clinical Dementia Rating=0) older adults for up to four years. Standardized interviews were conducted to gather the circumstances of all reported falls, including the location, time of day, and activity at the time of the fall. We qualitatively analyzed fall interviews to determine the reason for the fall and factors of limited or divided attention preceding the fall. There were a total of 1,133 falls reported by 212 (61%) participants. The majority of falls occurred outdoors (53%), in the afternoon (45%), while walking or running (50%), and due to tripping over or catching feet on an object (37%). Additionally, 37% of falls occurred during instances of limited or divided attention. Taken together, these findings provide a greater understanding of the circumstances of older adult falls that may inform future classifications of types of falls and lead to tailored fall prevention approaches.

